# Editorial: RNA-chromatin interactions: biology, mechanism, disease, and therapeutics—volume 2

**DOI:** 10.3389/fgene.2024.1461574

**Published:** 2024-07-24

**Authors:** Malik Bisserier, Luciano G. Martelotto, Assam El-Osta, Prabhu Mathiyalagan

**Affiliations:** ^1^ Department of Cell Biology and Anatomy, Physiology New York Medical College, Valhalla, NY, United States; ^2^ Adelaide Centre for Epigenetics, South Australian ImmunoGenomics Cancer Institute, University of Adelaide, Adelaide, SA, Australia; ^3^ Baker Heart and Diabetes Institute, Epigenetics in Human Health and Disease Program, Melbourne, VIC, Australia; ^4^ Benthos Prime Central, Houston, TX, United States

**Keywords:** noncoding RNA, RNA, chromatin RNA, long noncoding RNA, epigenetics, miRNA, epitranscriptomics

## Introduction

RNA-based therapeutics have gained significant interest. Long noncoding RNAs (lncRNAs) interact with chromatin to regulate genome’s stabilization, gene transcription, X-chromosome inactivation, RNA-mediated epigenetic inheritance, DNA methylation, and activation and silencing of chromatin state maintenance (Statello et al., 2021). RNA-chromatin interactions occur at specific genomic loci through direct interactions between RNA and protein or RNA and double-stranded DNA (dsDNA) or both. Recent advances have highlighted the regulatory roles of lncRNA sequence features associated with specific RNA-protein interactions. The specificity of RNA-chromatin interactions is controlled by the unique sequence and structural features of lncRNAs and their epitranscriptomic modifications. Many lncRNAs are retained at specific genomic loci, exerting their function through selective interactions governed by their sequence, structural attributes, or epitranscriptomic marks, either alone or in combination. Additionally, the field of epitranscriptomics has revealed the importance of epitranscriptomic markers, such as m6A, m5C, and A-to-I RNA editing, which modulate lncRNA interactions with chromatin (He and He, 2021). However, our understanding of how these interactions are regulated and their biological implications remains poorly understood. It is critical to understand whether lncRNA function depends on their folding and is influenced by these sequence features and epitranscriptomic marks. Such insights could provide new structural clues to improve the therapeutic targeting of lncRNAs using antisense oligonucleotides or small-molecule inhibitors. Therefore, a better understanding of the unique features and mechanisms governing RNA-chromatin interactions is essential to identify new biomarkers and develop novel lncRNA-based therapeutics.

This volume 2, titled “RNA-Chromatin Interactions: Biology, Mechanism, Disease and Therapeutics,” emphasizes the importance of the RNA-chromatin code and aims to improve our understanding of how lncRNAs operate at the chromatin level to more accurately predict their binding partners during development and disease and to enhance the targeting of RNA-chromatin complexes for therapeutic purposes (Limouse et al., 2023; Oksuz et al., 2023). Moreover, it could help identify and map novel lncRNAs that exhibit context-dependent regulation and assign functions to genome-wide association studies to identify causal sequence variants that overlap lncRNAs. The articles in this volume explore the functional roles and mechanisms of RNA in disease and therapy but also underscore the growing importance of a focused RNA-centric view of chromatin in modern biological and medical research. Overall, the future of lncRNA research and its therapeutic applications relies on our ability to elucidate the complex interplay between lncRNA sequence, structure, and epitranscriptome and how these features individually or collectively stabilize RNA-chromatin interactions.

Among the key contributions in this volume, Sun et al. aimed to explore the underlying genetic factors of gallstone disease (GSD) by examining the expression profiles of lncRNAs and messenger RNAs (mRNAs). Using RNA sequencing on four paired human gallbladder samples, the researchers identified 934 differentially expressed mRNAs (DEmRNAs) and 304 differentially expressed lncRNAs (DElncRNAs). Functional enrichment analysis indicated their significant involvement in metabolic-related processes. Correlation analysis between DElncRNAs and DEmRNAs led to the construction of a co-expression network, as well as cis- and trans-regulatory networks. A competing endogenous RNA (ceRNA) network was also established. Validation through RT-qPCR on 10 paired samples confirmed the significant upregulation of AC004692.4, HECW1-IT1, SFRP4, and COMP, and downregulation of LINC01564, SLC26A3, RP1-27K12.2, and GSTA2 in gallstone samples, aligning with sequencing results. This comprehensive analysis enhances the understanding of RNA-based regulatory mechanisms in the GSD’s genetic etiology.

The review by Wang et al. highlights the growing importance of RNA epigenetic modifications, especially 5-methylcytosine (m5C), in cardiovascular diseases (CVD), which are projected to cause over 23 million deaths annually by 2030. It examines how m5C modifications in RNA influence cellular functions vital to cardiovascular health, such as RNA stability, export, translation, and stress response. These modifications, catalyzed by methyltransferases and reversed by demethylases, play crucial roles in mitochondrial function and stress responses. The review also discusses the roles of m5C modifications in heart failure, myocardial infarction, pulmonary hypertension, and atherosclerosis, elaborating on the molecular players (“writers,” “erasers,” and “readers”) involved in this regulation. The authors suggest that targeting the m5C methylation machinery could offer new therapeutic approaches for CVD.

Murshed et al. explored the crucial role of microRNAs (miRNAs) in the treatment of hepatocellular carcinoma, a major and deadly liver cancer. This highlights the significant progress in miRNA biology since its discovery in 1993, particularly its regulatory effects on gene expression, which are often disrupted in diseases such as HCC. This review article details the mechanisms of miRNA action, including their role in inhibiting mRNA translation and promoting mRNA degradation, thereby affecting cellular processes, such as proliferation and apoptosis. It also discusses the relationship between specific miRNAs and the progression of hepatocellular carcinoma by focusing on particular miRNAs as tumor suppressors and others as oncogenes. This review further examines the potential of miRNA-based therapies and explores innovative approaches, such as the use of miRNA mimics and inhibitors to regulate oncogenes and tumor suppressor genes. It addresses the challenges of miRNA therapy delivery, particularly the safety and targeting efficiency of viral and non-viral vectors, and speculates on future research directions and potential for clinical trials. This comprehensive review underlines the need for continued exploration of miRNA-based therapies in the fight against hepatocellular carcinoma, highlighting the potential for significant advances if delivery and specificity challenges can be overcome.

Another review by Wang et al. explores in detail the role of lncRNAs in tendon healing, emphasizing their regulatory functions in various stages of repair. lncRNAs modulate gene expression, influencing cellular processes such as inflammation, proliferation, and remodeling in tendon injury, healing, and repair. Key lncRNAs, including MALAT1, H19, and NEAT1, are highlighted for their contributions to tendon cell proliferation, migration, and differentiation. The authors reviewed the lncRNA literature carefully to propose that these lncRNAs exert their effects through various mechanisms, such as regulating fibroblast proliferation, tenogenic differentiation, and osteogenic differentiation of tendon-derived stem cells. At the molecular level, these lncRNAs interact with signaling pathways and transcription factors, thereby impacting extracellular matrix production and organization, promoting better cellular communication for efficient tissue repair. This review highlights the involvement of lncRNAs in tendon healing and underscores the therapeutic significance of lncRNAs in enhancing tendon repair outcomes and reducing complications such as scarring and adhesions.

## Implications and future directions

These studies provide insights into how RNA-chromatin interactions can influence disease development and therapeutic outcomes. This volume is not the end but rather the beginning of a series of articles we hope to expand in forthcoming issues. RNA-chromatin interactions play a central role in metabolic diseases like gallstone disease and more complex and multifactorial conditions like cardiovascular diseases and cancer. This volume advances our understanding of the specific roles of RNA within chromatin architecture but also opens new avenues for therapeutic interventions using RNA-based technologies. In conclusion, “RNA-Chromatin Interactions: Biology, Mechanism, Disease, and Therapeutics—Volume 2″provides essential insights into the complex roles of RNA within the chromatin and cellular environment in various diseases. With this new volume, we hope to pave the way for innovative therapeutic interventions by transforming how we approach disease diagnosis and treatment. Integrating these findings into clinical research is critical for developing effective RNA-based diagnostics and therapeutics as we continue to uncover these complex interactions.

## References

[B1] He P. C. He C. (2021). m(6) A RNA methylation: from mechanisms to therapeutic potential. EMBO J. 40, e105977. 10.15252/embj.2020105977 33470439 PMC7849164

[B2] Limouse C. Smith O. K. Jukam D. Fryer K. A. Greenleaf W. J. Straight A. F. (2023). Global mapping of RNA-chromatin contacts reveals a proximity-dominated connectivity model for ncRNA-gene interactions. Nat. Commun. 14, 6073. 10.1038/s41467-023-41848-9 37770513 PMC10539311

[B3] Oksuz O. Henninger J. E. Warneford-Thomson R. Zheng M. M. Erb H. Vancura A. (2023). Transcription factors interact with RNA to regulate genes. Mol. Cell. 83, 2449–2463 e13. 10.1016/j.molcel.2023.06.012 37402367 PMC10529847

[B4] Statello L. Guo C. J. Chen L. L. Huarte M. (2021). Gene regulation by long non-coding RNAs and its biological functions. Nat. Rev. Mol. Cell. Biol. 22, 96–118. 10.1038/s41580-020-00315-9 33353982 PMC7754182

